# Amygdala activation during emotional face processing in adolescents with affective disorders: the role of underlying depression and anxiety symptoms

**DOI:** 10.3389/fnhum.2014.00393

**Published:** 2014-06-05

**Authors:** Bianca G. van den Bulk, Paul H. F. Meens, Natasja D. J. van Lang, E. L. de Voogd, Nic J. A. van der Wee, Serge A. R. B. Rombouts, Eveline A. Crone, Robert R. J. M. Vermeiren

**Affiliations:** ^1^Department of Child and Adolescent Psychiatry, Curium-Leiden University Medical CenterOegstgeest, Netherlands; ^2^Brain and Development Lab, Department of Developmental and Educational Psychology, Institute of Psychology, Leiden UniversityLeiden, Netherlands; ^3^Leiden Institute for Brain and Cognition, Leiden UniversityLeiden, Netherlands; ^4^Department of Developmental Psychology, Faculty of Social and Behavioral Science, University of AmsterdamAmsterdam, Netherlands; ^5^Department of Psychiatry, Leiden University Medical CenterLeiden, Netherlands; ^6^Department of Radiology, Leiden University Medical CenterLeiden, Netherlands

**Keywords:** adolescence, depression, anxiety, face processing, amygdala, individual differences

## Abstract

Depressive and anxiety disorders are often first diagnosed during adolescence and it is known that they persist into adulthood. Previous studies often tried to dissociate depressive and anxiety disorders, but high comorbidity makes this difficult and maybe even impossible. The goal of this study was to use neuroimaging to test what the unique contribution is of depression and anxiety symptomatology on emotional processing and amygdala activation, and to compare the results with a healthy control group. We included 25 adolescents with depressive and/or anxiety disorders and 26 healthy adolescents. Participants performed an emotional face processing task while in the MRI scanner. We were particularly interested in the relation between depression/anxiety symptomatology and patterns of amygdala activation. There were no significant differences in activation patterns between the control group and the clinical group on whole brain level and ROI level. However, we found that dimensional scores on an anxiety but not a depression subscale significantly predicted brain activation in the right amygdala when processing fearful, happy and neutral faces. These results suggest that anxiety symptoms are a better predictor for differentiating activation patterns in the amygdala than depression symptoms. Although the current study includes a relatively large sample of treatment naïve adolescents with depression/anxiety disorders, results might be influenced by differences between studies in recruitment strategies or methodology. Future research should include larger samples with a more equal distribution of adolescents with a clinical diagnosis of depression and/or anxiety. To conclude, this study shows that abnormal amygdala responses to emotional faces in depression and anxiety seems to be more dependent on anxiety symptoms than on depression symptoms, and thereby highlights the need for more research to better characterize clinical groups in future studies.

## Introduction

Depressive and anxiety disorders often have their onset during adolescence; adolescent prevalence rates for mood disorders are estimated to be around 10%, and rates for any anxiety disorders are as high as 24.9% (Kessler et al., 2012) and Costello et al. (2011) found a continued increase in the prevalence of depressive and anxiety disorders from adolescence to adulthood. It is also known that the comorbidity between depressive and anxiety disorders during adolescence is high. For example, within a clinical group of adolescents with a depression diagnosis, almost half also had an anxiety disorder (Essau, 2008). Comorbidity carries clinical relevance because it predicts a more negative outcome: Lewinsohn et al. (1995) reported a poorer global functioning in a comorbid depressed-anxious group than in the “pure” depression group.

Elucidating the underlying neurobiological mechanisms of these disorders may be crucial to fully understand the negative outcomes in adolescent depression and anxiety. fMRI studies in particular can be helpful for relating disturbed psychological and neurobiological processes with clinical severity. A better relationship between neuroscience and clinical research may result in better diagnoses and increased understanding in the future (Pine et al., 2008). However, studies investigating underlying neurobiological mechanisms generally focus on adolescents with a specific disorder, without fully taking comorbidity and dimensionality into account (e.g., Brotman et al., 2007; McClure et al., 2007; Monk et al., 2008b; Yang et al., 2010; Perlman et al., 2012; Strawn et al., 2012). In the current study we included a group of adolescents with depression and/or anxiety disorders and investigated the neurobiological correlates of emotional face processing with special regard for the individual differences and symptom dimensionality of these disorders.

Adolescents with depressive or anxiety disorders are known to show similar disturbed emotion perception (Thomas et al., 2001) and regulation (Bar-Haim et al., 2007; Shechner et al., 2012). In the last decade, a number of task-based fMRI studies have focused on the brain mechanisms related to emotion processing in depressed and anxious adolescents (e.g., Thomas et al., 2001; McClure et al., 2007; Monk et al., 2008a,b; Perlman et al., 2012). In these studies, participants are often asked to passively view, label, or rate emotional faces. For instance, in a study by Mingtian et al. (2012) adolescents were asked to recognize and match faces by emotional expression, while in other studies, they were asked to interpret emotional faces by focusing their attention to their own internal state or to other more external objectives in the face (i.e., McClure et al., 2007). In these studies, the amygdala is consistently reported to play an important role in the processing of emotional faces (Fusar-Poli et al., 2009; Whalen et al., 2009). The amygdala is part of the social information processing network and the overlapping face processing network (Scherf et al., 2012). It is known that the amygdala plays an important role in learning associations between a stimulus and its emotional significance (Tottenham et al., 2009). Prior research indicated that amygdala activity increases in response to both positive and negative face stimuli (Davis and Whalen, 2001; Somerville et al., 2004; van den Bulk et al., 2013). Meta-analyses show that the amygdala is most strongly activated for fearful and disgusted faces and to a somewhat lesser extent for happy and neutral faces (Costafreda et al., 2008; Fusar-Poli et al., 2009). However, amygdala activation not only depends on emotional valence but also on the cognitive demands of a paradigm. For example, explicit face processing (e.g., directing attention to emotional features of the face) increases bilateral amygdala activation relative to implicit face processing (e.g., diverting attention to nose width; Fusar-Poli et al., 2009). Also, amygdala activation decreases when participants are instructed to label faces or to indicate their own subjective feeling compared to a passive viewing condition (Costafreda et al., 2008). Overall, the amygdala is strongly involved in emotion processing and an important brain area for underlying neural correlates of depressive and anxiety disorders.

Studies investigating the neurobiological correlates of emotional face processing in adolescents with depressive disorders have found inconsistent associations with amygdala activation (Monk, 2008; Hulvershorn et al., 2011). For example, a study by Thomas et al. (2001) showed blunted amygdala response to fearful faces in a group of adolescent girls with a major depressive disorder, while two other studies showed heightened amygdala response in mixed gender groups with a major depressive disorder (Roberson-Nay et al., 2006) or youths at high risk for depression (Monk et al., 2008a). In contrast, studies in anxious adolescents were much more consistent, as multiple studies reported heightened amygdala responses to fearful and angry faces (Thomas et al., 2001; McClure et al., 2007). For example, a study by Monk et al. (2008b), in which they scanned youths with generalized anxiety disorder, showed heightened patterns of amygdala activation in response to briefly presented masked angry faces. Based on these studies, differentiating patterns of amygdala activation during face processing tasks seems to be related to depressive and anxiety disorders and it might indicate an underlying neurobiological mechanism of depression and anxiety. However, it is not yet completely clear what the unique contribution of depression or anxiety is too these differentiating activation patterns.

Only a few clinical studies in adolescents investigated the relation between amygdala activation and symptom severity (Thomas et al., 2001) or the difference in patterns of amygdala activation between depressed and anxious adolescents (e.g., Beesdo et al., 2009) when studying emotional face processing. For example, Thomas et al. (2001) correlated daily self-reported anxiety with amygdala activation in adolescents with depressive or anxiety disorders. They found a significant positive correlation between daily reported anxiety and activation in the amygdala. More recently, Beesdo et al. (2009) reported common patterns of amygdala activation between adolescents with depression and adolescents with anxiety during active fearful face processing (focused attention on internally experienced fear). However, during passive viewing the anxious adolescents showed hyperactivation of the amygdala while depressed adolescents showed hypoactivation. These results indicate that there are common and distinct neural patterns of amygdala activation between depression and anxiety that might be explained by task design or disorder-specifics characteristics.

There are also a few studies that investigated the relation between self-reported levels of anxiety (within the normal range) and amygdala activation in non-clinical adolescent or (young) adult samples (e.g., Monk et al., 2003; Somerville et al., 2004; Stein et al., 2007; Ball et al., 2012). In general the results of these studies showed a positive relation between levels of anxiety and amygdala activation suggesting that levels of anxiety influence amygdala activation.

Although there are some studies that investigated the relation between depression, anxiety, and amygdala activation in both clinical and non-clinical samples, more research is necessary to further delineate the unique contributions of depression and anxiety symptomatology to differentiating patterns of amygdala activation during face processing. This will aid in understanding individual differences between adolescents who have (comorbid) depressive or anxiety disorders and how they perceive and regulate negative, neutral and positive emotions. Also, using a dimensional approach is in line with the Research Domain Criteria approach (Insel et al., 2010), which is intended to provide a new classification framework for research into psychopathology. Therefore, the purpose of the present study is to investigate the underlying neurobiological correlates of emotional face processing in treatment-naïve adolescents with a depressive and/or anxiety disorders and in matched healthy controls. We were specifically interested in whether there is a relation between severity of depression or anxiety symptoms and activation patterns in the amygdala within a comorbid depression/anxiety group. This creates the opportunity to investigate depression and anxiety dimensionally instead of only using a categorical distinction between the two disorder groups. Based on previous studies we expected heightened patterns of amygdala activation in the clinical group compared to the control group. Based on prior findings by Thomas et al. (2001), we also expected a strong positive relation between self-reported anxiety symptoms and amygdala activation (see also Monk et al., 2003; Somerville et al., 2004; Stein et al., 2007; Ball et al., 2012).

## Methods

### Participants

Functional MRI data were collected for 25 treatment naïve adolescents with a clinical diagnoses of a current DSM-IV depressive or anxiety disorder [Mean Age _(*SD*)_ = 15.44_(1.53)_, 21 females] and 26 healthy controls [Mean Age_(_*_SD_*_)_ = 14.65_(1.55)_, 23 females]. The sex distribution was unequal with a higher number of females than males due to the focus on internalizing disorders, which occur more often in females than in males. Within the clinical group 17 adolescents were diagnosed with a depressive disorder, six with an anxiety disorder and two with an adjustment disorder with depression and anxiety characteristics (see Table 1). All adolescents took part in the larger EPISCA study (Emotional Pathways' Imaging Study in Clinical Adolescents).

**Table 1:** **Group characteristics for the clinical and control group**.

	**Clinical group (***N*** = **25**)**		**Control group (***N*** = **26**)**		
	***N***		***N***		***p***
Females	21/4		23/3		n.s.
	**Mean**	***SD***	**Mean**	***SD***	***p***
Age	15.44	1.53	14.65	1.55	n.s.
Full scale IQ	105	8.73	106	7.77	n.s.
**Clinical DSM-IV diagnoses**	***N***	**%**			
Depression	7	13.7			
Dysthymia	10	19.8			
GAD	3	5.9			
SAD	2	3.9			
Adjustment disorder with dep./anx.	2	3.9			
Anxiety disorder NOS	1	2			
**CDI**^†^	**Mean**	***SD***	**Mean**	***SD***	***p***
Total score	18.86	9.24	4.56	3.40	*p* < 0.001
RCADS^*^	**Mean**	***SD***	**Mean**	***SD***	***p***
Total of five anxiety scale scores	31.65	14.46	14.85	12.83	*p* < 0.001

† CDI questionnaire data was missing for one participant with an adjustment disorder with depression characteristics, resulting in N = 24 for the total sample.

* *RCADS anxiety subscale questionnaire data was missing for three participants (one with an adjustment disorder with depression and anxiety characteristics and two with a depressive disorder) resulting in N = 22 for the total sample*.

The adolescents from the clinical group were recruited in outpatient departments of two child and adolescent psychiatric institutes. The inclusion criteria were: having a clinical diagnosis of any depression or anxiety disorder, being referred for regular CBT-like psychotherapy, and being treatment naïve. They were excluded when other primary diagnoses were present or when they used psychotropic medications. The healthy control group adolescents were recruited through local advertisement, with the following inclusion criteria: no clinical scores on validated mood and behavioral questionnaires, no history of traumatic experiences and no current psychotherapeutic intervention of any kind. All participants met the following inclusion criteria: aged between 12 and 19, estimated full scale IQ ≥ 80, right-handed, normal, or corrected-to-normal vision, sufficient understanding of the Dutch language, no history of neurological impairments and no contraindications for MRI testing.

For all participants, estimated full-scale IQ scores were acquired with six subtests of the Wechsler Intelligence Scale for Children-III or the Wechsler Adult Intelligence Scale (Wechsler, 1991, 1997). Both the clinical group [Mean_(_*_SD_*_)_ = 105_(8.73)_] and the control group [Mean_(_*_SD_*_)_ = 106_(7.63)_] scored in the average range. Overall, there were no significant differences between the groups considering age [*F*_(1, 49)_ = 2.73, *p* = 0.105], estimated full scale IQ [*F*_(1, 49)_ = 0.368, *p* = 0.547] and sex [χ^2^(1) = 0.214, *p* = 0.642], and all participants were drug- and treatment naive.

Ten additional participants (clinical *N* = 5, control *N* = 5) were excluded from the analyses due to: unforeseen clinical features (*N* = 1 control), use of medication (SSRI's; *N* = 1 clinical), technical problems during scanning (*N* = 3 clinical, *N* = 1 control), excessive head movement (>3 mm, *N* = 2 control) or anomalous findings reported by the radiologist (*N* = 1 clinical, *N* = 1 control).

Informed consent was obtained by participants, and by parents and participants in case of minors. The adolescents received a financial compensation including travel expenses for participation. The medical ethics committee of the Leiden University Medical Centre approved the study and all anatomical scans were reviewed and cleared by a radiologist.

### Clinical assessment

Participants of the clinical group were included if they were diagnosed with any current DSM-IV depressive or anxiety disorder following clinical assessment by a child- and adolescent psychiatrist. Categorical DSM-IV diagnoses were further assessed with the Anxiety Disorders Interview Schedule (ADIS) for children and parents (Silverman and Albano, 1996). In addition, standardized dimensional measures were used for assessing the severity of self-reported symptoms of depression and anxiety; i.e., the Children's Depression Inventory (CDI; Kovacs, 1992) and the Revised Child Anxiety and Depression Scale (RCADS; Chorpita et al., 2000). The CDI is a self-report questionnaire with 27 items that correspond with dimensions of DSM-IV depressive disorders, and is scored on a 3-point Likert scale (0 = *absence of symptomatology* to 2 = *severe symptomatology*). The RCADS is a self-report questionnaire with 47 items that correspond with dimensions of DSM-IV depressive and anxiety disorders. The items are descriptive statements that are scored on a 4-point Likert scale (0 = *never* to 3 = *always*). In the current study, we only used the total score of the five RCADS anxiety scales. For the control group, the same clinical instruments were used. Control group adolescents were excluded when they fulfilled the criteria for a DSM-IV diagnosis (ADIS interview) or had sub-clinical scores on clinical questionnaires. Both questionnaires showed high levels of internal consistency: alpha for the CDI total scale 0.94 and for the RCADS anxiety subscale was 0.95.

### Task

We administered an emotional faces task that was originally developed by McClure et al. (2007; Monk et al., 2003) and that showed robust differences in brain activation patterns between participants with and without internalizing disorders. We described the adaptations we made in detail previously (van den Bulk et al., 2013). In short, the task consisted of three constrained (questions: “how afraid are you?,” “how happy are you?,” and “how wide is the nose?”) and one unconstrained (passive viewing) attention condition. After condition presentation, participants viewed 21 emotional faces (fearful, neutral or happy facial expression with an equal distribution of male and female actors) per attention condition, which they had to rate on a four-point rating scale [(1) not at all; (2) a little; (3) quite; and (4) very]. During the task, reaction times and subjective scorings of the different emotional faces (fearful, happy, or neutral) were recorded for behavioral analyses.

**Figure 1:**
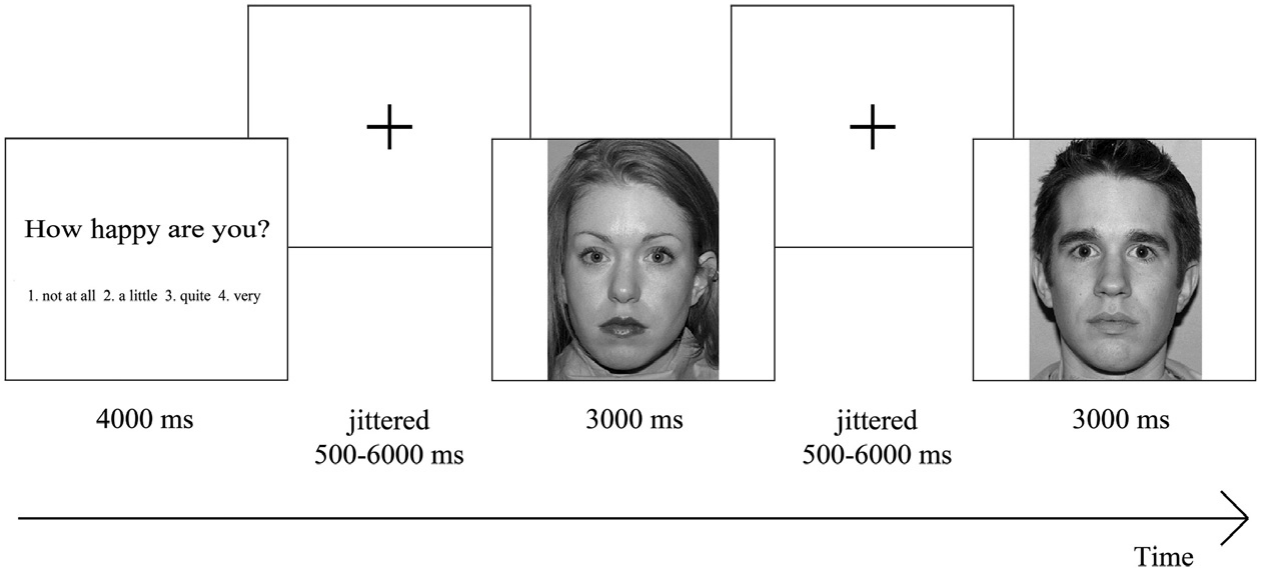
**Overview of task design**. Participants were presented with an attention condition, followed by a centrally located fixation cross. Thereafter, they saw one of the emotional faces, again followed by a centrally located fixation cross, after which another emotional face was shown. Participants had to rate each emotional face on a four-point rating scale ranging from “not at all” to “very,” based on the presented attention condition.

All trials had the same structure: first participants were presented with one of the attention conditions for 4000 ms which was followed by a centrally located fixation cross with a jittered interval between 500 and 6000 ms. Thereafter, one of the pictures was shown for 3000 ms again followed by a centrally located fixation cross (Figure1). Nothing happened when participants did not respond within 3000 ms and those trials were recorded as missing trials (1.53% in total), which were not included in the analyses. We are aware of the ongoing debate whether the term “neutral” faces exist, or whether the term “ambiguous” faces should be used (e.g., Tahmasebi et al., 2012), but for consistency with our previous paper we use the term “neutral” faces.

### Image acquisition

Data were acquired using a 3.0T Philips Achieva (Philips, Best, The Netherlands) scanner at the Leiden University Medical Centre. Stimuli were presented onto a screen located at the head of the scanner bore and viewed by participants by means of a mirror mounted to the head coil assembly. First, a localizer was obtained for each participant. Subsequently, T2^*^-weighted Echo-Planar Images (EPI) (*TR* = 2.2 s. *TE* = 30 ms, 80 × 80 matrix, FOV = 220, 38 slices of thickness 2.72 mm) were obtained during three functional runs of 192 volumes each. Each run had two additional scans at the start that were discarded to allow for equilibration of T1 saturation effects. Also, a sagittal 3-dimensional gradient-echo T1-weighted image was acquired for registration purposes with the following scan parameters: repetition time = 9.8 ms; echo time = 4.6 ms; flip angle = 8°; 140 sagittal slices; no slice gap; FOV = 224; 1.17 × 1.17 × 1.20 mm voxels; duration 4:56 min.

### Behavioral analyses

The effects of emotional faces on subjective scoring were examined for each attention condition separately, using group (2 levels) × emotion (3 levels) repeated measurement ANOVAs in SPSS 19. The scores were analyzed separately for each attention condition, because values of the scores represent different interpretations for each condition. For reaction time, one repeated measurement ANOVA was performed with a group (2 levels) by emotion (3 levels) design. In case sphericity was not assumed, Greenhouse-Geisser correction (GG-corr.) was applied. No outliers were detected in the task data and the questionnaire data.

### fMRI analyses

We used SPM8 (Welcome Department of Cognitive Neurology, London) to analyze the acquired data. Data was preprocessed using the following steps: (1) realignment of functional time series to compensate for small head movements and differences in slice timing acquisition; (2) registration and normalization of functional volumes (from EPI to individual structural T1 and thereafter to the T1 template); (3) spatial smoothing of the functional volumes with an 8 mm, full-width at half-maximum isotropic Gaussian kernel. The normalization algorithm used a 12-parameter affine transformation together with a non-linear transformation involving cosine basis functions and resampled the volumes to 3 mm. cubic voxels. The MNI (Montreal Neurological Institute) 305 stereotaxic space templates (Cocosco et al., 1997) were used for visualization and all results are reported in this template, which is an approximation of Talairach space (Talairach and Tournoux, 1988).

Individual subjects' data were analyzed using the general linear model in SPM8. The fMRI time series were modeled by a series of events convolved with a canonical hemodynamic response function (HRF). The attention conditions were modeled separately as 4000 ms events and were added as covariates of no interest. The picture presentation of each emotional face was modeled as a zero duration event. In the model, the picture presentation was further divided in 12 separate function trials (four attention conditions by three expressed emotions). The modeled events were used as a covariate in a general linear model along with a basic set of cosine functions that high-pass filtered the data. The least squares parameter estimates of the height of the best-fitting canonical HRF for each condition were used in pair wise contrasts (e.g., all faces vs. fixation and fearful faces vs. fixation). The resulting contrast images, computed on a subject-by-subject basis, were submitted to group analyses. At the group level, we performed a full factorial model in which we included a factor called condition (12 levels, condition > null contrasts) and a factor called group (2 levels). We were mainly interested in the main effect of group, the interaction effect of group × condition and the overall task effects. Task-related responses were considered significant if they consisted of at least 10 contiguous voxels at a corrected threshold of *p* < 0.05 (FDR corrected).

We used the MarsBaR toolbox for use with SPM8 (http://marsbar.sourceforge.net/; Brett et al., 2002) to perform region of interest (ROI) analyses to further investigate patterns of activation. ROIs were defined based on a priori hypothesis about the bilateral amygdala (anatomically defined). No outliers were detected in the ROI output.

### Correlation and regression analyses

To examine the relation between symptom severity and amygdala activation patterns, we correlated scores of the anxiety scale of the RCADS and the total CDI score with the ROI percent signal change values of the whole anatomically defined amygdala in SPSS. Furthermore, we performed stepwise regression analyses with percent signal change values as a dependent variable and the demeaned scores of the RCADS anxiety scale, CDI total scale and an interaction term of these two as independent variables. The correlation and regression analyses were performed for each emotion separately, collapsed across attention conditions, resulting in three regression analyses. There were no outliers in the data (i.e., deviating >3 standard deviations) and expectation maximization was used when items in the RCADS (3 in total) and CDI (6 in total) were missing.

## Results

### Behavioral data

#### Subjective rating

**Figure 2:**
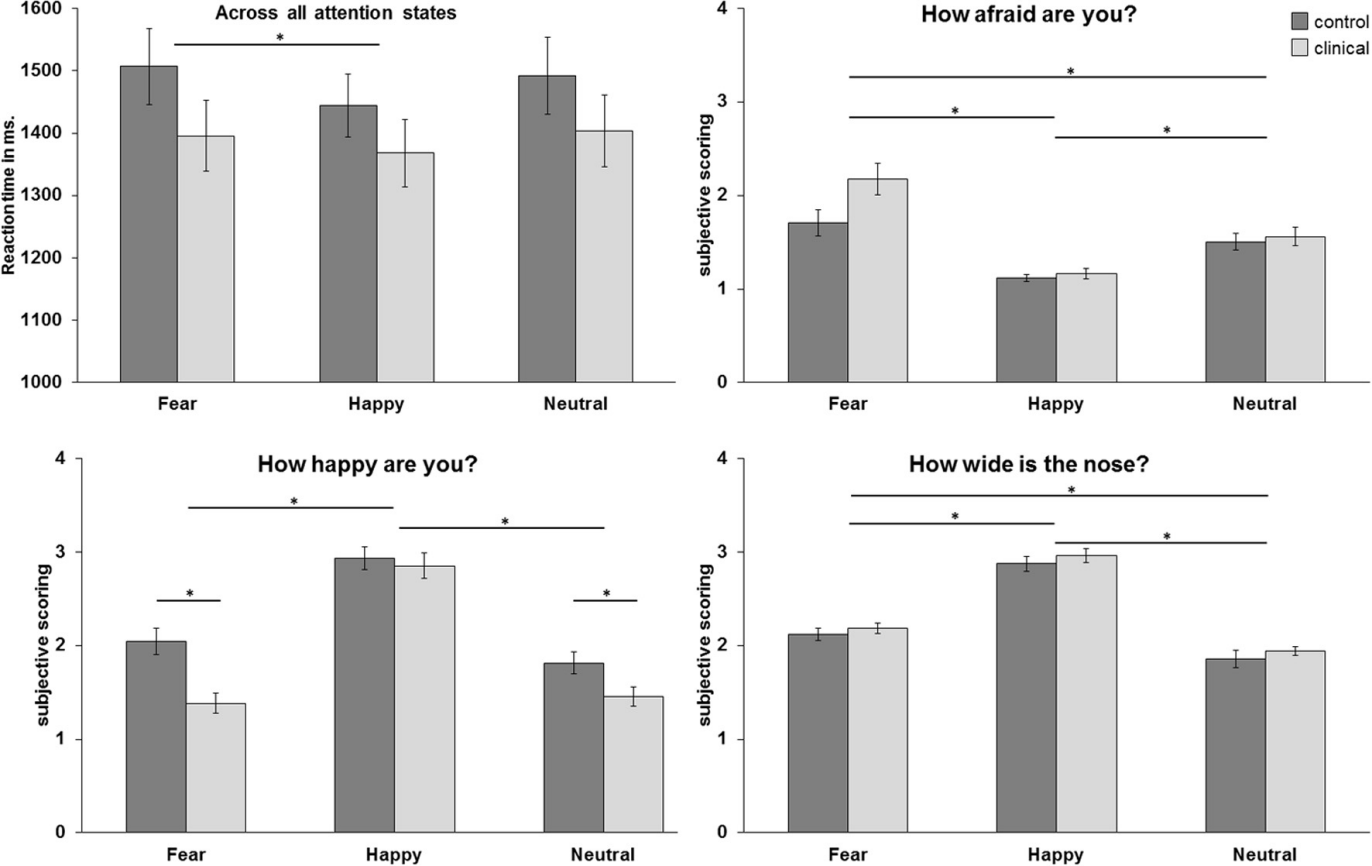
**Mean reaction times in milliseconds across all attention conditions and mean subjective scoring per attention condition**. ^*^p < 0.05.

The repeated measurement ANOVA for the condition “how afraid are you?” resulted in a main effect of emotion [*F*_(2, 98)_ = 36.66, *p* < 0.001, GG-corr.], with higher subjective scorings for fearful faces than for neutral and happy faces (resp. *p* < 0.005 and *p* < 0.001) and higher scorings for neutral faces compared to happy faces (*p* < 0.001). Furthermore, there was a trend for an interaction effect between emotion and group [*F*_(2, 98)_ = 3.33, *p* = 0.054, GG-corr.], with higher scorings for fearful faces in the clinical group compared to the control group. The ANOVA for the condition “how happy are you?” resulted in a main effect of emotion [*F*_(2, 98)_ = 100.53, *p* < 0.001, GG-corr.] and a main effect for group [*F*_(1, 49)_ = 8.44, *p* < 0.01]. Furthermore, this ANOVA resulted in an emotion x group interaction [*F*_(2, 98)_ = 4.24, *p* < 0.05, GG-corr.] in which the clinical group gave lower ratings to fearful and neutral faces than the control group (*p* < 0.001 and*p* < 0.05, respectively). Finally, the ANOVA for the condition “how wide is the nose?” resulted in a main effect for emotion [*F*_(2,98)_ = 331.39, *p* < 0.001], with higher subjective scoring for happy faces than for fearful and neutral faces (both *p*'s < 0.001). Also, subjective scoring was higher for fearful faces than for neutral faces (*p* < 0.001). There was no main effect of group or an interaction effect with group in this condition. See also Figure 2 for an overview of the behavioral effects.

#### Reaction Times

The ANOVA for reaction times resulted in a main effect for emotion [*F*_(2,98)_ = 4.04, *p* < 0.05], with higher reaction times for fearful faces than for happy faces (*p* = 0.05).

### Whole brain analyses

**Figure 3:**
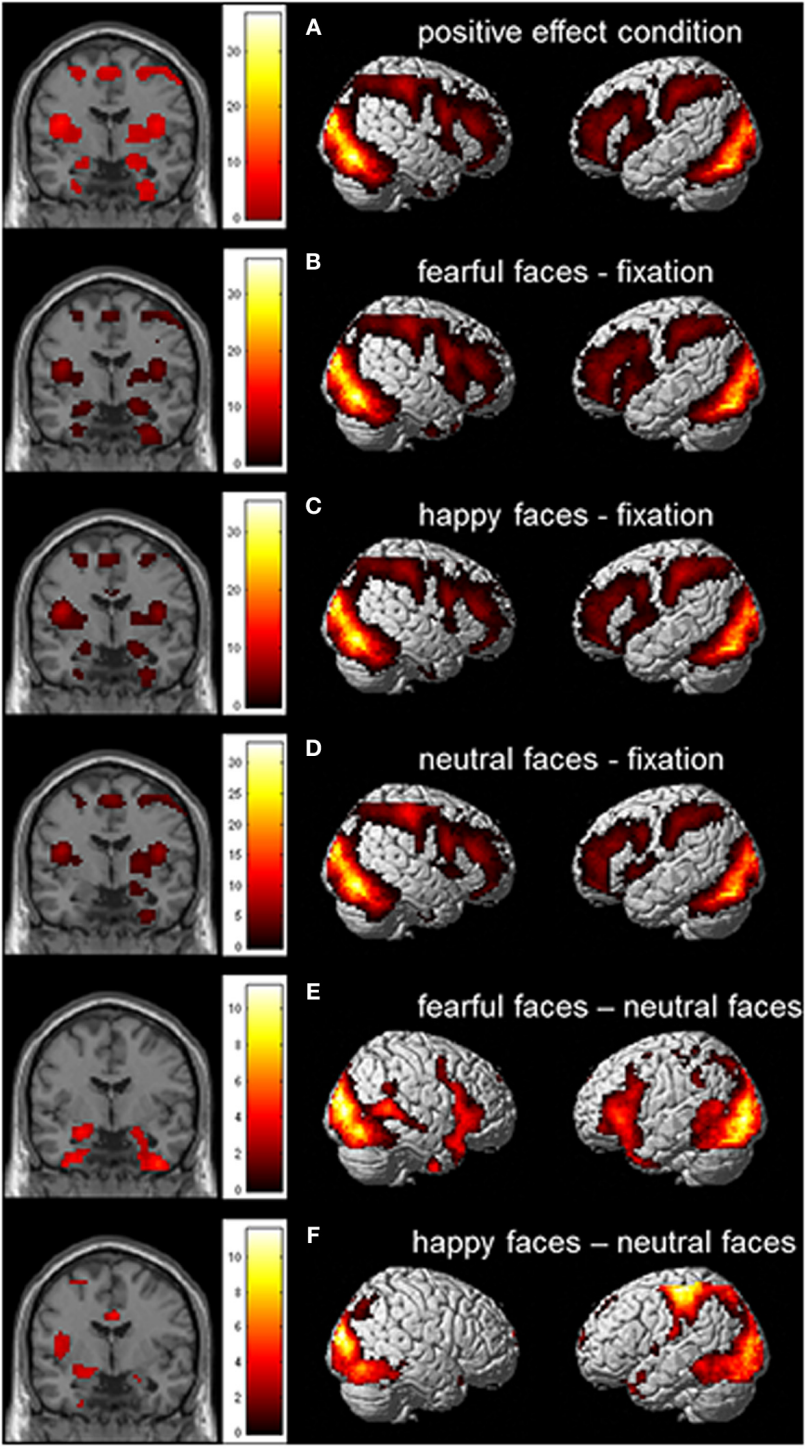
**Whole brain contrast showing effects for (A) positive effect of condition; (B) fearful faces > fixation; (C) happy faces > fixation; (D) neutral faces > fixation; (E) fearful faces > neutral faces; and (F) happy faces > neutral faces (*N* = 51; FDR corrected, *p* < 0.05; 10 contiguous voxels)**. MNI coordinate coronal slices: *x* = 21, *y* = −4, *z* = −17.

We first performed whole brain analyses to examine whether the task activated brain regions that were previously found to be related to emotional face processing. The whole brain Omnibus ANOVA for the positive effect of condition showed activation in bilateral amygdala, bilateral insula and bilateral prefrontal cortex (PFC; see also Figure 3A). To further investigate the task effect we created the contrasts fearful faces > fixation, happy faces > fixation and neutral faces > fixation (Figures 3B–D). These contrasts resulted in activation in (bilateral) amygdala, bilateral insula, and bilateral PFC. Finally we created the contrasts fearful faces > neutral faces en happy faces > neutral faces. These contrasts resulted in activation in the bilateral amygdala, bilateral uncus and bilateral inferior frontal gyrus/insula for fearful faces > neutral faces and activation in the left amygdala, left insula en medial prefrontal cortex for happy faces > neutral faces (Figures 3E,F; see also Table S1).

To examine whether the amygdala specifically was responsive to emotional valence and not to attention condition (as expected based on our previous study, van den Bulk et al., 2013), we also created the contrasts fear rating > passive viewing, happy rating > passive viewing, nose rating > passive viewing, fear rating > nose rating, and happy rating > nose rating. The analyses showed that all active conditions resulted in more activation in bilateral PFC compared to the passive viewing condition. Importantly, amygdala activation was not modulated by attention condition.

Furthermore, we examined the main effect of group and the interaction effect between group and condition. These contrasts showed no significant patterns of brain activation for the main effect of group and for the interaction effect between group and condition, suggesting that there are no significant differences in whole brain activation between groups and for all conditions.

### Region of interest analyses

**Figure 4:**
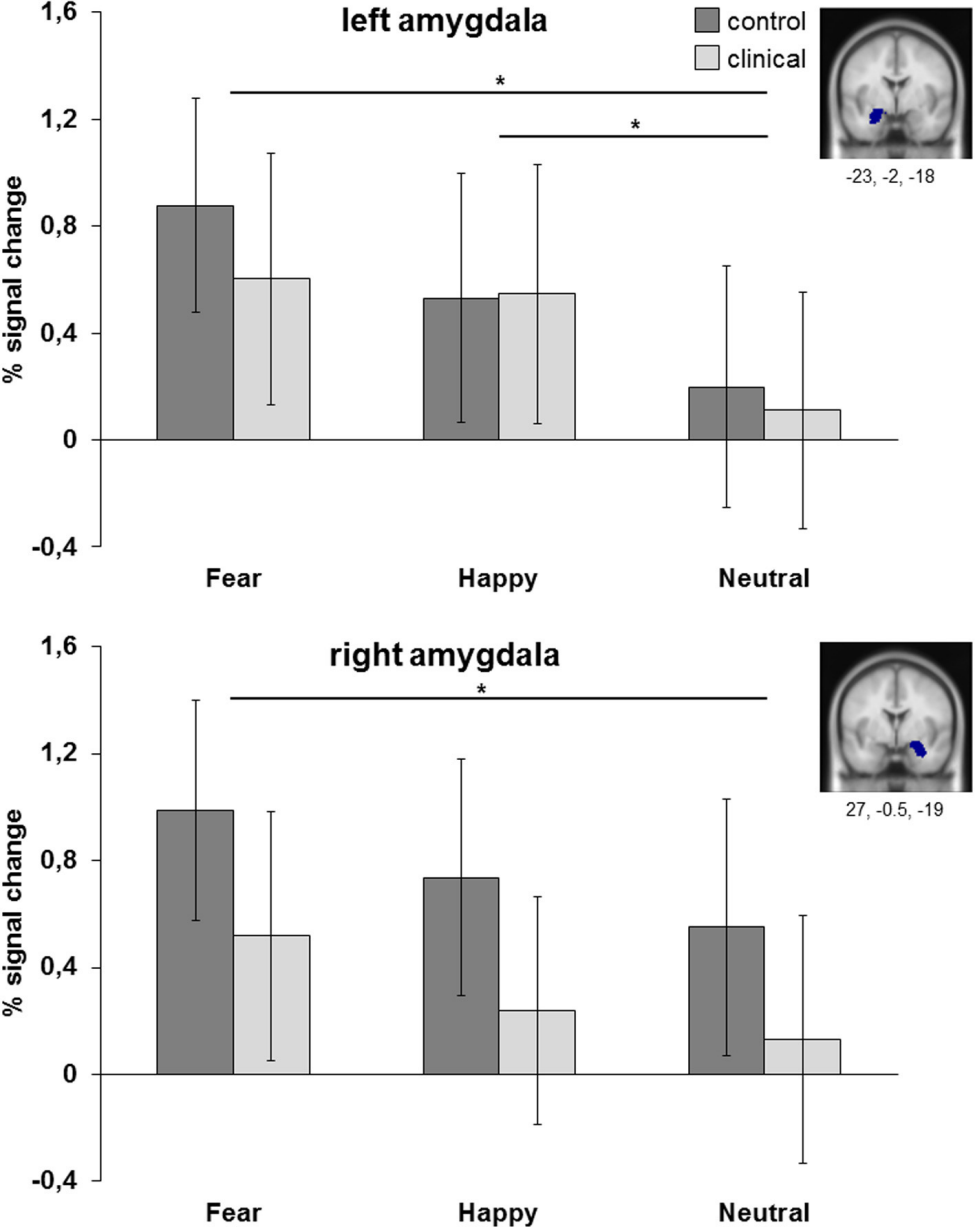
**ROI analyses of left and right amygdala (anatomical)**. Results are collapsed across attention conditions. ^*^p < 0.05.

The analyses presented above were followed up by ROI analyses allowing us to detect smaller changes in specific regions that do not survive whole-brain comparisons, thereby allowing for a more detailed test of potential group differences. Results are reported for anatomically defined amygdala ROIs, based on the MNI templates available in SPM (see Figure 4). The percent signal change values of the left and right ROI were submitted to attention condition (4 levels) × emotion (3 levels) × group (2 levels) ANOVAs. Results were highly comparable for the masked functional amygdala ROIs, based on the contrast all faces > fixation, FDR corrected, *p* < 0.05, at least 10 continues voxels.

The ANOVA for left amygdala resulted in a main effect of emotion [*F*_(2, 98)_ = 10.09, *p* < 0.001, η^2^_partial_ = 0.171]. *Post-hoc* analysis showed that amygdala was more active for fearful and happy faces than for neutral faces (resp. *p* = 0.001 and *p* < 0.01), while fearful and happy faces did not differ from each other (*p* = 0.40). Comparable results were obtained for the right amygdala, with a main effect of emotion [*F*_(2, 98)_ = 5.66, *p* = 0.005, η^2^_partial_ = 0.104] resulting in more amygdala activation to fearful faces than to neutral faces (*p* < 0.05). There were no main or interaction effects with group. For both regions there was no main effect of group [left: *F*_(1, 49)_ = 0.11, *p* = 0.74, η^2^_partial_ = 0.002; right: *F*_(1, 49)_ = 1.57, *p* = 0.22, η^2^_partial_ = 0.031], no interaction effect between group and attention condition [left: *F*_(3, 147)_ = 0.62, *p* = 0.60, η^2^_partial_ = 0.013; right: *F*_(3, 147)_ = 1.21, *p* = 0.31, η^2^_partial_ = 0.024] and no interaction effect between group and emotion [left: *F*_(2, 98)_ = 0.61, *p* = 0.54, η^2^_partial_ = 0.012; right: *F*_(2, 98)_ = 0.05, *p* = 0.95, η^2^_partial_ = 0.001].

### Relation between depression/anxiety symptoms and amygdala activation

**Figure 5:**
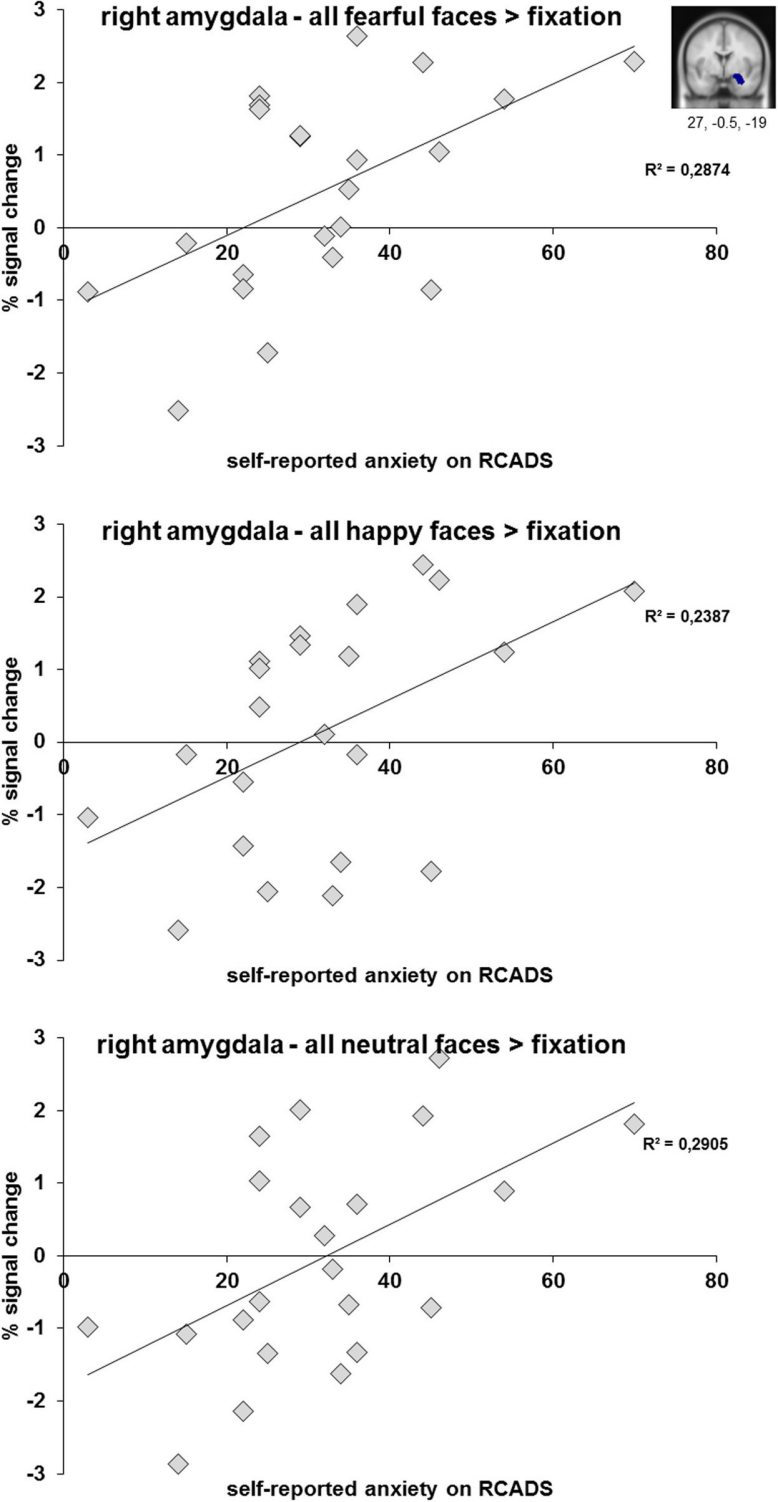
**Scatter plots of the correlation analyses between right amygdala activation (anatomical ROI) during the processing of fearful, happy, and neutral faces and subjective scoring on the anxiety subscale of the RCADS**. Results are presented for *N* = 22 adolescents with a depressive and/or anxiety disorder.

When correlating the percent signal change values of the amygdala ROIs (separately for fearful, happy and neutral faces relative to fixation) with anxiety (RCADS) and depression symptoms (CDI), we only found significant positive correlations between right amygdala activation and self-reported anxiety for fearful, happy, and neutral faces relative to fixation in the clinical group (Figure 5). The significant correlations ranged between *r* = 0.49 and *r* = 0.54, all with *p* < 0.05 (see also Table S2). We found no significant correlations for self-reported depression symptoms in the clinical group (all *p*'s ≥ 0.10). Furthermore, we found no significant correlations between amygdala activation and anxiety or depression symptomatology for the complete sample (*N* = 51; all *p*'s ≥ 0.17) and the control group (all *p*'s ≥ 0.21).

We performed three linear regression analyses for *N* = 22 adolescents from the clinical group with percent signal change of the amygdala ROI (for fearful, happy, and neutral relative to fixation separately) as dependent variable and demeaned anxiety score (RCADS), depression score (CDI score; both in model I) and the interaction between both (in model II) as independent variables. For the regression analyses in which we included amygdala activation when viewing fearful faces, the results showed that model I explained 29% of variance [*R*^2^ = 0.290, *F*_(2, 21)_ = 3.884, *p* < 0.05] and that the anxiety scores significantly predicted amygdala activation when viewing fearful faces (β = 0.509, *p* < 0.05). Model II, in which the interaction between anxiety and depression scores was included, did not result in an increase in explained variance and was not significant. When performing the same analyses with amygdala activation when viewing neutral faces as dependent variable, model I explained 29.5% of variance [*R*^2^ = 0.295, *F*_(2, 21)_ = 3.977, *p* < 0.05] and again the anxiety scores predicted amygdala activation (β = 0.499, *p* < 0.05). In the analysis for happy faces model I explained 23.9% of variance [*R*^2^ = 0.239, *F*_(2, 21)_ = 2.991, *p* = 0.074] which was at trend level and anxiety scores predicted amygdala activation (β = 0.480, *p* = 0.057).

Analyses reported were also performed while including age in years in step one and when excluding males. The results of these analyses were highly comparable with the results reported here.

## Discussion

The objective of this study was to investigate emotional face processing in a sample of treatment naïve adolescents with a depression or anxiety diagnosis. In this sample with comorbid depression and anxiety symptoms we investigated the contribution of self-reported dimensional depression and anxiety scores to specific patterns of amygdala activation. The emotional face processing task activated expected brain regions in the emotional face processing network (e.g., bilateral amygdala, bilateral insula, and bilateral PFC). Contrary to prior reports (Thomas et al., 2001; Roberson-Nay et al., 2006; McClure et al., 2007; Monk et al., 2008a,b), we found no differences between the clinical group and the control group in the whole brain analyses or in the more specific ROI analyses of the amygdala. However, consistent with other prior studies (e.g., Thomas et al., 2001) we found strong positive correlations within the clinical group between levels of self-reported anxiety symptoms and right amygdala activation, for all three types of emotional valence (i.e., fearful, happy, and neutral face processing). Interestingly, there were no significant relations between amygdala activation and self-reported depression symptoms. Follow-up regression analyses confirmed that levels of self-reported anxiety were predictive for right amygdala activation. These correlation and regression effects were not present in the complete sample of *N* = 51 and not in the control group. This suggests that the relation between self-reported anxiety symptomatology and amygdala activation may be specific for adolescents with depressive/anxiety disorders. Furthermore, it might indicate that there was not enough variance within the scores of the control group to find significant correlations.

The whole brain results showed bilateral amygdala activation to fearful, happy and neutral faces, with a stronger response to fearful and happy faces in the left amygdala and to fearful faces only in the right amygdala. These results correspond to the existing literature in which heightened patterns of amygdala activation are often reported when viewing negative emotional faces (see for an overview Davis and Whalen, 2001; Costafreda et al., 2008). Notably, researchers have shown that the amygdala also responds to positive emotional faces (e.g., Somerville et al., 2004; Fusar-Poli et al., 2009; van den Bulk et al., 2013). The results of our study correspond to these findings and support prior conclusions that the amygdala is more of a general emotion processing node than only a negativity/fear processing node (Whalen, 1998; Cunningham et al., 2008).

Previous studies in which adolescents with a clinical depression or anxiety disorder were included, reported differentiating patterns of amygdala activation in the clinical group when they were compared with a control group (Thomas et al., 2001; Roberson-Nay et al., 2006; McClure et al., 2007; Monk et al., 2008a,b). Yet, in the current study we could not replicate these results: the clinical adolescents did not show significantly differentiating patterns of amygdala activation on whole brain or ROI level. The absence of this effect was present in contrasts in which we used fixation as baseline condition and in which we used neutral faces as baseline condition. One of the reasons that we did not find group differences in amygdala activation may be due to the changes we made to the original task (see van den Bulk et al., 2013) or differences in recruitment between studies. For example, we chose to include stimuli of fearful, happy and neutral facial expressions and not of angry and sad facial expressions. Also, we used direct gaze instead of averted gaze, only one head orientation (straight) and we asked the participants to focus on their own subjective experience during face viewing. It might be that the use of other task designs (e.g., rating of arousal or valence; see for an overview Costafreda et al., 2008; Sauer et al., 2013) results in different findings.

Furthermore, prior studies have differed by including adolescents with only a specific depressive or anxiety disorder. We included adolescents with various clinical diagnoses of affective disorders in our study, as we feel that taking a more dimensional approach is more ecologically valid given the frequent comorbidity between depressive and anxiety disorders and symptomatology (e.g., Essau, 2008). Furthermore, previous research indicated that depression is often (72% of cases in community setting and 62% of cases in clinical setting) preceded by an anxiety disorder (Essau, 2008), which also highlights the tight relation between these disorders. By including a combined group and by taking the comorbidity of symptomatology into account we were able to investigate the specificity of the underlying mechanisms in both depressive and anxiety disorders. We think that this is a better approach to these clinical disorders.

Although we did not find a significant difference in amygdala activation between groups, we were still interested in the unique contribution of self-reported depression and anxiety symptoms to amygdala activation. That is to say, an individual difference analysis may be more sensitive for detecting heightened amygdala activation, as this may be present more in those adolescents with most severe problems. When taking the dimensional perspective of symptom severity into account, we found a significant positive relation between levels of self-reported anxiety and amygdala activation in the clinical group, which is in line with previous clinical studies (e.g., Thomas et al., 2001). The current findings suggest that the level of anxiety symptoms, and not depression symptoms, seems to be a good predictor for differentiating patterns of amygdala activation independent of clinical disorder/diagnosis. This in turn might suggest that during adolescence anxiety symptoms and the relation with amygdala activation is an underlying trait characteristic for both depression and anxiety disorders and that depression symptoms are more a state characteristic. In the current study, adolescents who score high on anxiety symptomatology show more amygdala activation independent of emotional valence. It might be that these adolescents show a heightened vigilance in general and not only for scary or frightening situations. In future research this should be further investigated by, for example, also collecting data on personality traits like “neuroticism” or by applying the state trait anxiety inventory (STAI; Spielberger et al., 1983). Research already indicated that both state and trait anxiety highly relates to neuroticism (del Barrio et al., 1997; Kotov et al., 2010) and it would be interesting to further investigate the relation between state and trait anxiety symptoms, neuroticism, and differentiating patterns of amygdala activation. When translating the current findings to our understanding of the symptoms belonging to anxiety and when taking the absence of the effect in the control group into account, it might be that there is a predisposition for the development of depression or anxiety that may be expressed by personality styles like neuroticism. However, these ideas are highly speculative and further research is necessary to investigate this. For example, it would be interesting to see whether children/young adolescents who score high on neuroticism earlier in life have a higher chance of developing depression and/or anxiety during adolescence/young adulthood and to see whether this relates to differentiating patterns of amygdala activation.

There are some limitations in the current study that should be mentioned. First, even though the sample size of both our groups (*N* = 25 clinical adolescents and *N* = 26 controls) is relatively large compared to other studies (e.g., Thomas et al., 2001; Monk et al., 2008b) it might have been too small to find robust group differences. In addition, including a larger group of adolescents with clinical depression and anxiety disorders would be helpful to examine the relationship between anxiety and depression symptoms with amygdala activation in more detail. Therefore, future studies should aim for a more equal distribution between adolescents with DSM-IV depression and anxiety diagnosis and larger sample sizes to better isolate the relative contributions of depression and anxiety symptoms on a dimensional scale. Second, the age range of the participating adolescents was quite broad. Previous research has indicated that amygdala activation might be influenced by development, since children, adolescents and adults show different activation patterns when viewing emotional faces (Hare et al., 2008; Somerville et al., 2011). Even though we did not find age effects in our study, future studies should include adolescents within a smaller age range and ideally use multiple adolescent groups with slightly different ages to examine the developmental pattern of amygdala activation in both clinical and non-clinical adolescents. Within these future studies it would also be interesting to investigate the effect of puberty in relation to depressive and anxiety disorders, symptomatology, and amygdala activation. Levels of progesterone, which relate to the menstrual cycle in females, influence patterns of amygdala activation during emotional face processing (Derntl et al., 2008). This probably also relates to puberty, and maybe even to depression or anxiety symptomatology. In the current study we did collect information about puberty stages (not about menstrual cycle), but the majority of adolescents already met post-puberty criteria and there was not enough variability within the sample to perform valid analyses. Finally, our sample included more female than male participants, which might have influenced our results. However, it is known that depressive and anxiety disorders are much more common in females than in males, which might underline the clinical validity of our sample. Nevertheless, it would be interesting to examine sex differences on the functioning of the amygdala related to face processing in future studies.

To conclude, the current study revealed that levels of self-reported anxiety were associated with patterns of amygdala activation for different types of emotional faces and across clinical diagnoses. Our findings thereby confirmed our hypothesis that anxiety symptoms are related to amygdala activity, but disconfirmed the hypothesis that clinical groups in general are different from healthy control participants. As such, a dimensional perspective seems to be a better approach for differentiating patterns of brain activation than categorical division of clinical vs. non-clinical adolescents. In future research, it will be important to include longitudinal measurements to further investigate the relation between symptomatology, amygdala activation and treatment outcome in adolescents. Also, it would be interesting to include functional connectivity analyses to examine the relation between differentiating patterns of amygdala activation and connectivity with other brain regions, for example PFC. These suggestions are in line with the research Domain Criteria approach in which a dimensional approach is considered important to advance understanding of mental disorders (Insel et al., 2010). Extending our knowledge on these topics, can give more information about individual differences in treatment outcome. The results of those studies can set the stage for the development of new diagnosis and treatment guidelines for adolescent depressive and anxiety disorders. This study is a first step in this process by highlighting the need for more research to better characterization of participant groups in future studies.

## Author contributions

Author Bianca G. van den Bulk collected data, managed literature searches, performed analyses and wrote the manuscript. Authors Paul H. F. Meens and E. L. de Voogd also collected data and were involved in data analyses. Authors Nic J. A. van der Wee, Natasja D. J. van Lang, Serge A. R. B. Rombouts and Robert R. J. M. Vermeiren designed the study and author Natasja D. J. van Lang wrote the protocol and was involved in literature searches. Author Eveline A. Crone was involved in data management, data analyses and manuscript writing. Furthermore, all authors were involved in task design. All authors contributed to the paper by critically review and revise the paper and they all have approved the final manuscript.

### Conflict of interest statement

The authors declare that the research was conducted in the absence of any commercial or financial relationships that could be construed as a potential conflict of interest.

## Supplementary material

Table S1 **Whole brain activation patterns for the contrasts: (A) positive effect of condition; (B) fearful faces > fixation; (C) happy faces > fixation; (D) neutral faces > fixation; (E) fearful faces > neutral faces; and (F) happy faces > neutral faces.** Coordinates represent significant peaks of activation at *p* < 0.05, FDR-corrected, 10 contiguous voxels and are listed in MNI space. ^*^*p* < 0.05 when corrected for multiple comparisons at cluster-level (FWE).

Table S2 **Pearson correlations between anxiety (RCADS) and depression (CDI) subscale scores and parameter estimates of left and right amygdala ROI values for ***N*** = **22** adolescents from the clinical group.**
