# Supplementary material for: Amygdala activation during emotional face processing in adolescents with affective disorders: the role of underlying depression and anxiety symptoms
Source: Front Hum Neurosci. 2014 Jun 5;8:393. doi: 10.3389/fnhum.2014.00393 (PMC4046490; doi:10.3389/fnhum.2014.00393)
Supplement: Table S1 — Whole brain activation patterns for the contrasts: (A) positive effect of condition; (B) fearful faces > fixation; (C) happy faces > fixation; (D) neutral faces > fixation; (E) fearful faces > neutral faces; and (F) happy faces > neutral faces. Coordinates represent significant peaks of activation at p < 0.05, FDR-corrected, 10 contiguous voxels and are listed in MNI space. *p < 0.05 when corrected for multiple comparisons at cluster-level (FWE). [file DataSheet1.ZIP › Supp Table 1.DOCX]

**Supplemental table 1.** Whole brain activation patterns for the contrasts: A. all faces > fixation, B. fearful faces > fixation, C. happy faces > fixation, D. neutral faces > fixation, E. Regions represent significant peaks of activation at *p*<.05, FDR-corrected, 10 contiguous voxels and coordinates are listed in MNI space and represent peak values. * = *p*<.05 when corrected for multiple comparisons at cluster-level (FWE).

| **Contrast** | **Region** | **Side** | **z-score** | **K*_E_*** | **x** | **y** | **z** |  |
| --- | --- | --- | --- | --- | --- | --- | --- | --- |
| **A.** |  |  |  |  |  |  |  |  |
| **Positive effect condition** | Superior frontal gyrus | L | Inf. | 776 | 0 | 11 | 58 | * |
|  | Middle frontal gyrus | L | Inf. | 2377 | -51 | 38 | 28 | * |
|  | Middle frontal gyrus | L | 3.53 | 43 | -27 | -4 | 58 |  |
|  | Inferior frontal gyrus | R | 7.65 |  | 48 | 47 | 7 |  |
|  | Medial frontal gyrus | L | Inf. |  | -3 | 17 | 49 |  |
|  | Postcentral gyrus | L | Inf. | 1285 | -51 | -28 | 49 | * |
|  | Postcentral gyrus | L | 5.48 |  | -57 | -19 | 25 |  |
|  | Precuneus | L | 6.50 |  | -27 | -61 | 52 |  |
|  | Superior temporal gyrus | R | 2.89 |  | 30 | 17 | -41 |  |
|  | Superior temporal gyrus | R | 2.71 |  | 42 | 20 | -38 |  |
|  | Middle occipital gyrus | R | Inf. |  | 15 | -91 | 13 |  |
|  | Lingual gyrus | L | Inf. |  | -3 | -82 | -5 |  |
|  | Lingual gyrus | R | Inf. | 12221 | 9 | -79 | -5 | * |
|  | Cingulate gyrus | L | 3.02 | 17 | -3 | 5 | 28 |  |
|  | Insula | L | Inf. |  | -39 | -4 | 16 |  |
|  | Thalamus | L | 7.26 | 76 | -21 | -28 | -2 |  |
|  | Parahippocampal gyrus | L | 3.26 | 33 | -21 | -7 | -17 |  |
|  | Uncus | L | 3.75 | 32 | -30 | -10 | -35 |  |
|  | Uncus | R | 6.86 | 117 | 30 | -10 | -38 |  |
| **B.** |  |  |  |  |  |  |  |  |
| **Fearful faces –fixation** | Superior frontal gyrus | L | Inf. | 768 | 0 | 11 | 58 | * |
|  | Middle frontal gyrus | L | Inf. | 2858 | -51 | 38 | 28 | * |
|  | Middle frontal gyrus | L | 3.15 | 24 | -27 | -4 | 58 |  |
|  | Inferior frontal gyrus | L | Inf. |  | -48 | 47 | 7 |  |
|  | Medial frontal gyrus | L | Inf. |  | -3 | 17 | 49 |  |
|  | Middle temporal gyrus | L | 2.66 | 10 | -48 | 14 | -38 |  |
|  | Inferior occipital gyrus | R | Inf. |  | 33 | -76 | -11 |  |
|  | Lingual gyrus | L | Inf. |  | -3 | -82 | -5 |  |
|  | Lingual gyrus | R | Inf. | 13667 | 9 | -79 | -5 | * |
|  | Insula | L | 7.35 |  | -39 | -4 | 16 |  |
|  | Thalamus | L | 7.20 | 71 | -21 | -28 | -2 |  |
| **C.** |  |  |  |  |  |  |  |  |
| **Happy faces - fixation** | Superior frontal gyrus | L | Inf. | 712 | 0 | 11 | 58 | * |
|  | Middle frontal gyrus | L | Inf. |  | -51 | 38 | 28 |  |
|  | Inferior frontal gyrus | L | 7.06 |  | -48 | 50 | 7 |  |
|  | Medial frontal gyrus | L | Inf. |  | -3 | 17 | 49 |  |
|  | Precentral gyrus | L | 3.06 |  | -39 | -10 | 64 |  |
|  | Cingulate gyrus | L | 3.63 | 45 | -3 | 5 | 28 |  |
|  | Cingulate gyrus | R | 3.01 |  | 6 | -1 | 31 |  |
|  | Cuneus | L | Inf. |  | -9 | -94 | 4 |  |
|  | Middle occipital gyrus | R | Inf. | 13308 | 15 | -91 | 13 | * |
|  | Lingual gyrus | R | Inf. |  | 9 | -82 | -5 |  |
|  | Cerebellar tonsil | L | 3.62 | 10 | -24 | -40 | -41 |  |
|  | Insula | L | Inf. | 2304 | -39 | -4 | 16 | * |
|  | Thalamus | L | Inf. | 120 | -21 | -28 | -2 |  |
|  | Uncus | L | 3.68 | 33 | -30 | -10 | -35 |  |
|  | Uncus | R | 6.70 | 98 | 30 | -10 | -38 |  |
| **D.** |  |  |  |  |  |  |  |  |
| **Neutral faces – fixation** | Superior frontal gyrus | L | Inf. |  | 0 | 11 | 58 |  |
|  | Middle frontal gyrus | L | 7.70 | 1450 | -51 | 38 | 28 | * |
|  | Middle frontal gyrus | L | 3.22 | 20 | -21 | 26 | -20 |  |
|  | Middle frontal gyrus | L | 3.03 | 18 | -24 | -4 | 55 |  |
|  | Inferior frontal gyrus | L | 5.89 |  | -48 | 47 | 7 |  |
|  | Inferior frontal gyrus | L | 5.75 |  | -60 | 11 | 37 |  |
|  | Medial frontal gyrus | L | Inf. | 760 | -6 | 20 | 49 | * |
|  | Postcentral gyrus | L | 7.59 | 1057 | -54 | -25 | 49 | * |
|  | Precuneus | L | 5.10 |  | -27 | -61 | 52 |  |
|  | Cuneus | L | Inf. |  | -3 | -82 | -5 |  |
|  | Inferior parietal lobule | L | 6.91 |  | -45 | -38 | 46 |  |
|  | Lingual gyrus | L | Inf. |  | -15 | -79 | -11 |  |
|  | Lingual gyrus | R | Inf. | 11767 | 9 | -79 | -5 | * |
|  | Insula | L | 7.20 | 504 | -39 | -4 | 16 | * |
|  | Insula | L | 5.14 |  | -42 | 14 | 1 |  |
|  | Insula | L | 5.11 |  | -30 | 20 | 7 |  |
|  | Thalamus | L | 5.48 | 44 | -21 | -28 | -2 |  |
|  | Uncus | R | 6.19 | 70 | 30 | -10 | -38 |  |
| **E.** |  |  |  |  |  |  |  |  |
| **Fearful faces – neutral faces** | Middle occipital gyrus | L | Inf. | 7539 | -30 | -88 | 4 | * |
|  | Superior frontal gyrus | R | 3.09 | 14 | 9 | 59 | 40 |  |
|  | Middle frontal gyrus | L | 2.95 | 13 | -39 | 2 | 61 |  |
|  | Inferior frontal gyrus | L | 4.90 |  | -48 | 29 | -2 |  |
|  | Inferior frontal gyrus | R | 5.29 | 1177 | 57 | 23 | 4 | * |
|  | Inferior frontal gyrus | R | 3.97 |  | 60 | 20 | 25 |  |
|  | Postcentral gyrus | L | 2.78 | 36 | -48 | -22 | 61 |  |
|  | Postcentral gyrus | L | 2.76 |  | -45 | -40 | 64 |  |
|  | Postcentral gyrus | L | 2.57 |  | -36 | -31 | 49 |  |
|  | Cingulate gyrus | R | 2.88 | 10 | 15 | 23 | 37 |  |
|  | Inferior parietal lobule | L | 3.23 | 61 | -57 | -40 | 40 |  |
|  | Superior temporal gyrus | L | 4.45 |  | -39 | 23 | -26 |  |
|  | Inferior temporal gyrus | R | 4.69 |  | 39 | -4 | -44 |  |
|  | Middle occipital gyrus | R | Inf. |  | 30 | -88 | 10 |  |
|  | Lingual gyrus | L | Inf. |  | 0 | -76 | -2 |  |
|  | Parahippocampal gyrus | L | 4.94 | 1737 | -27 | 2 | -17 | * |
|  | Supramarginal gyrus | L | 3.12 |  | -60 | -46 | 34 |  |
| **F.** |  |  |  |  |  |  |  |  |
| **Happy faces – neutral faces** | Superior frontal gyrus | L | 3.27 | 63 | -6 | 68 | 22 |  |
|  | Superior frontal gyrus | L | 3.18 |  | -21 | 68 | 13 |  |
|  | Superior frontal gyrus | L | 2.96 | 25 | -15 | 44 | 49 |  |
|  | Superior frontal gyrus | L | 2.68 |  | -15 | 53 | 40 |  |
|  | Postcentral gyrus | L | Inf. |  | -42 | -25 | 64 |  |
|  | Cingulate gyrus | R | 3.93 | 28 | 3 | -4 | 34 |  |
|  | Anterior cingulate | L | 2.76 | 11 | 0 | 17 | 22 |  |
|  | Anterior cingulate | R | 2.69 |  | 3 | 8 | 28 |  |
|  | Cuneus | L | Inf. |  | -12 | -91 | 1 |  |
|  | Superior temporal gyrus | L | 3.45 | 69 | -42 | 11 | -20 |  |
|  | Superior temporal gyrus | L | 3.39 |  | -39 | 23 | -29 |  |
|  | Superior temporal gyrus | L | 3.15 | 18 | -27 | 11 | -38 |  |
|  | Superior temporal gyrus | R | 2.89 | 13 | 36 | 17 | -26 |  |
|  | Middle temporal gyrus | R | 2.95 | 11 | 72 | -34 | -8 |  |
|  | Middle occipital gyrus | R | Inf. | 8824 | 19 | -91 | 13 | * |
|  | Parahippocampal gyrus | L | 3.57 | 246 | -24 | -1 | -14 |  |
|  | Uncus | L | 3.05 |  | -24 | 2 | -38 |  |
|  | Subcallosal gyrus | L | 3.11 |  | -12 | 11 | -14 |  |
|  | Subcallosal gyrus | R | 3.33 |  | 3 | 14 | -17 |  |
