# Supplementary material for: Amygdala activation during emotional face processing in adolescents with affective disorders: the role of underlying depression and anxiety symptoms
Source: Front Hum Neurosci. 2014 Jun 5;8:393. doi: 10.3389/fnhum.2014.00393 (PMC4046490; doi:10.3389/fnhum.2014.00393)
Supplement: Table S1 — Whole brain activation patterns for the contrasts: (A) positive effect of condition; (B) fearful faces > fixation; (C) happy faces > fixation; (D) neutral faces > fixation; (E) fearful faces > neutral faces; and (F) happy faces > neutral faces. Coordinates represent significant peaks of activation at p < 0.05, FDR-corrected, 10 contiguous voxels and are listed in MNI space. *p < 0.05 when corrected for multiple comparisons at cluster-level (FWE). [file DataSheet1.ZIP › Supp Table 2.DOCX]

**Supplemental table 2.** Pearson correlations between anxiety (RCADS) and depression (CDI) subscale scores and parameter estimate of left and right amygdala ROI values for N=22 adolescents from the clinical group.

|  | **CDI**  **depr.** | **RCADS anx** |
| --- | --- | --- |
| CDI - depr. | - | - |
| RCADS - anx | .534** | - |
| l amygdala fearful | .203 | .167 |
| l amygdala happy | .082 | .112 |
| l amygdala neutral | .330 | .262 |
| r amygdala fearful | .324 | .537** |
| r amygdala happy | .274 | .489* |
| r amygdala neutral | .342 | .539** |

*=*p*<.05, **=*p*≤.01
